# Medical Education

**Published:** 1866-04

**Authors:** 


### EDITORIAL AND MISCELLANEOUS.

---

### MEDICAL EDUCATION.

No subject connected with the Profession of Medicine has engaged the attention of physicians more, for several years prior to the late war, than that which heads this article. Since the war is over, and the din of battle has subsided, this war of words amongst medical journalists, professors of colleges and physicians generally, is being reinaugurated.

A somewhat extended article on this subject appears in the editorial department of the Richmond *Medical Journal* for March. From the earnest and spirited manner in which the editor leads off, we were flattered with the hope that some certain and practical plan to insure more thorough and perfect medical education, was about to be suggested; but after perusing the article carefully, we must say, that, while we are pleased with the object in view, and the zeal with which its accomplishment is sought, we find no suggestion for carrying into effect the proposed change, which will probably lead to more practical results than those mentioned heretofore.

All must desire to see students of medicine well educated in the science before they are authorized to assume the responsibilities of the profession, and the fact that the standard of requirements for the degree, in many of the medical colleges, is too low, is felt and deplored by all true lovers of medical science; and yet such wholesale censure as the Richmond *Journal* indulges toward teachers and institutions not Trans-Atlantic, we think will not meet with favorable response from the profession generally. Many of us claim our *Alma Mater* this side the water, and are proud of the advancement made in the medical sciences by men whose success is due to teachings in American Colleges. The means by which to insure efficiency in those assuming the duties of the physician, is a problem, for the solution of which, certain members of the profession have been engaged for years. Lengthening the term of study; increasing the number of courses to be taken; adding to the usual number of chairs; confining the courses to winter months; increasing the length of the interval between the courses, &c., &c., have, in turn, been mentioned as the means through which the desired improvement must come. A practical and feasible plan is hinted at in the article above alluded to—one which was advocated years ago by the Atlanta *Medical and Surgical Journal*—and we think strikes at the root of any short coming that colleges may be inclined to indulge. If, through self-interest, sympathy, or any other cause, colleges allow ignorant men to graduate in medicine, a board of eminent and honorable members of the profession may be organized, unconnected with teaching and not liable to the prejudices of selfish influences, so much complained of as the sources of this crying evil. If a bona fide effort to raise the standard of requirements for graduation be intended, let the independent board be organized in every State, whose especial and only duty it shall be to decide upon the qualification of applicants for the degree; then, and not until then, will there be a higher standard uniformly observed. If, as is said, selfish interests direct faculties in their demands upon students, under the present system, we suggest, that instead of thus securing patronage by the ease with which the degree is obtained, let the thorough preparation and certainty of success of the applicants furnished the independent board, be made the means of accomplishing those ends. Then will colleges become directly and pecuniarily interested in adopting the best system of instruction.

The laity of the profession may unite, as suggested by the Richmond *Medical Journal*, in a demand upon the colleges to furnish better educated physicians, and the penalty—that of withholding patronage on failure to comply—may be thrust before their eyes at every corner of the street, and yet the desideratum is not attained. Each college claims that its alumni are equal to those of any other; and how is the proof controverting it to be produced and the verdict rendered? "What is everybody's business, is nobody's business;" and this community of physicians is not likely to inaugurate any systematic plan of ferreting out delinquents. Nor will the united sentiment of the colleges on any of the propositions, touching the time of study required, intervals between the courses, number of teachers engaged, or the season of the year in which the session is held, affect at all the status of the graduate. Indeed, the greater the requirements and the more stringent the exactions for admission to examination, the less so will they be in the qualification for the Degree.

The length of time occupied in preparing for the medical profession does not guarantee thoroughness of preparation to all. Some in every medical class are thoroughly prepared to commence, on their own responsibility, the practical study of medicine---at the bedside after the usual course of study; while others are not, nor would they be in double the time, should the terrorum of stringent exactions in the greenroom be withheld. Time alone cannot be depended on to insure competency, nor can long intervals between the courses of didactic study add to the probability of thorough preparation; but on the contrary, much is generally lost to the student, in the relaxation from study, and the recreation and pleasures generally indulged during the intervals.

This being the case—and we presume none will deny the truth of the statement—the shorter the interval the more thoroughly and perfectly will the student be educated in any given time. We have no reason to doubt the sincerity and good intentions of those, from whom the various plans have emanated, and many of the advocates doubtless have the honor and usefulness of the profession at heart. Then why may not all agree upon some particular arrangement by which the whole question may be disposed of in a manner satisfactory to the colleges, honorable to the profession, and beneficial to mankind? This everlasting quarrel among the colleges about the manner of teaching, the time af teaching, &c., can never effect one inch of movement in the desired direction. Let all agree to have a board as a general tribunal, with a member from each State having a medical college in it; or a board for each State—as above suggested—made of eminent resident physicians, unconnected with medical schools, whose duty it will be to pass upon the qualfications of all students presented by the colleges for the degree of Doctor of Medicine. The respectability, honor and pecuniary interest of the schools will, in this way, be placed in jeopardy by any failure to adopt the best mode of teaching to insure, in their pupils, the highest standard of qualification. That institution to which has been returned the largest number unfit to enter the profession, will, in self defense, seek to improve its system of instructions, and labor more assiduously to sustain a position which will warrant students in patronizing it with safety to their own interest.

These are the views we entertain of the principle by which these vexed questions may be settled, and without the adoption of some such mode, we predict incessant wrangling among teachers for all time to come, unless staid by the millennial reign.

